# Simulation of Nanopore Sequencing Signals Based on BiGRU

**DOI:** 10.3390/s20247244

**Published:** 2020-12-17

**Authors:** Weigang Chen, Peng Zhang, Lifu Song, Jinsheng Yang, Changcai Han

**Affiliations:** 1 School of Microelectronics, Tianjin University, Tianjin 300072, China; chenwg@tju.edu.cn (W.C.); zhangp@tju.edu.cn (P.Z.); jsyang@tju.edu.cn (J.Y.); 2 Frontier Science Center for Synthetic Biology (Ministry of Education), Tianjin University, Tianjin 300072, China; lifu.song@tju.edu.cn; 3 School of Chemical Engineering and Technology, Tianjin University, Tianjin 300072, China

**Keywords:** ONT nanopore sequencing, Bi-directional gated recurrent units, signal simulation, neural network

## Abstract

Oxford Nanopore sequencing is an important sequencing technology, which reads the nucleotide sequence by detecting the electrical current signal changes when DNA molecule is forced to pass through a biological nanopore. The research on signal simulation of nanopore sequencing is highly desirable for method developments of nanopore sequencing applications. To improve the simulation accuracy, we propose a novel signal simulation method based on Bi-directional Gated Recurrent Units (BiGRU). In this method, the signal processing model based on BiGRU is built to replace the traditional low-pass filter to post-process the ground-truth signal calculated by the input nucleotide sequence and nanopore sequencing pore model. Gaussian noise is then added to the filtered signal to generate the final simulated signal. This method can accurately model the relation between ground-truth signal and real-world sequencing signal through experimental sequencing data. The simulation results reveal that the proposed method utilizing the powerful learning ability of the neural network can generate the simulated signal that is closer to the real-world sequencing signal in the time and frequency domains than the existing simulation method.

## 1. Introduction

Nanopore-based sequencing technology is a new generation of sequencing technology, which has been rapidly developed in recent years [1,2]. It can detect the nucleotide sequences passing through the pores embedded in the membrane separating the two electrolyte chambers [3]. By applying a proper electric potential over the membrane, the nucleotide sequence can pass through the nanopore at an appropriate speed under the control of a DNA-translocating motor protein [4]. The bases in the pore change the nanopore conductance, causing changes in the current trace which can be used to determine the base types. Nanopore sequencing technology owns the advantages of long sequencing reads [5], real-time sequencing data analysis [6], and no PCR amplification [7]. In addition to nucleic acid sequencing, it also has great potential in virus sensing, protein sensing, and protein sequencing [8,9,10]. However, the weak signals on single-molecule level to be detected and relatively high complex sequencing environment will cause extremely noisy output signals, which is challenging for further base calling [11]. To solve this problem, a variety of digital signal analysis algorithms have been developed for nanopore sequencing applications [12,13,14]. Researchers usually use experimental data or simulated data to optimize and test the performance of these new algorithms [15]. Compared with experimental data, simulated data can greatly save the cost of research, reduce the difficulty of data analysis, and improve the efficiency of research. Therefore, more accurate simulation of nanopore sequencing data is highly desirable for method developments of nanopore sequencing technology.

The simulation methods of nanopore sequencing data can be divided into two categories: nanopore sequencing read simulation and nanopore sequencing signal simulation. ReadSim, SiLiCO, and NanoSim [16] are sequencing read simulators that generate the simulated reads by utilizing the input nucleotide sequence and configuration file, where the configuration file contains a set of parameters, such as insertion rate, deletion rate, substitution rate, read length, etc. The main difference among these three read simulators is that ReadSim uses the fixed configuration file, SiLiCO uses the configuration file provided by the user, and NanoSim uses the experimental data to learn the configuration file to be used in the simulation stage. DeepSimulator [17,18] is the first simulator that can simulate nanopore sequencing signals. Firstly, it generates a ground-truth signal corresponding to the input nucleotide sequence according to the nanopore sequencing pore model provided by Oxford Nanopore Technology (ONT) and the repeat time distribution of real sequencing signals. The ground-truth signal is an idealized simulated signal without any randomization. Next, it adopts a low-pass filter to filter out the high-frequency components embedded in the ground-truth signal that are not related to the real signal. Finally, to make the filtered signal without noise characteristic closer to the noisy nanopore raw signal, Gaussian noise is added to the filtered signal to generate the final simulated signal. Since the low-pass filter attenuates all high-frequency components that are higher than the cutoff frequency, it will inevitably attenuate the high-frequency components related to the real signal. For some input nucleotide sequences, there may be a large deviation between the simulated signals and the real signals, which causes inconvenience to the users who are concerned about the signal outputs.

To further improve the simulation accuracy of Oxford Nanopore sequencing signals, we propose a signal simulation method based on Bi-directional Gated Recurrent Units (BiGRU). For the convenience of description, we named the proposed method NanosigSim (https://github.com/zpllx/NanosigSim). BiGRU is a type of artificial neural network, which has been widely used in the field of basecalling to model the sequencing raw signal data. Specifically, by building a signal processing model based on BiGRU instead of using a low-pass filter, the ground-truth signal calculated from the input nucleotide sequence and pore model can be filtered more accurately, and then the final simulated signals can be generated by adding Gaussian noise to the filtered signals. The proposed signal processing model has a novel architecture that couples a three-layer BiGRU and a fully connected layer. This enables it to model the relationship between ground-truth signals and real-world sequencing signals accurately. Utilizing BiGRU neural network to learn the time-frequency characteristics of real-world sequencing signals from empirical data, the proposed method shows higher accuracy in the simulation of nanopore signals than DeepSimulator on four biological sequencing samples. The experimental data also verify the accuracy of the proposed signal simulation method regarding the time and frequency domains.

## 2. Materials and Methods

### 2.1. Main Workflow

**Figure 1:**
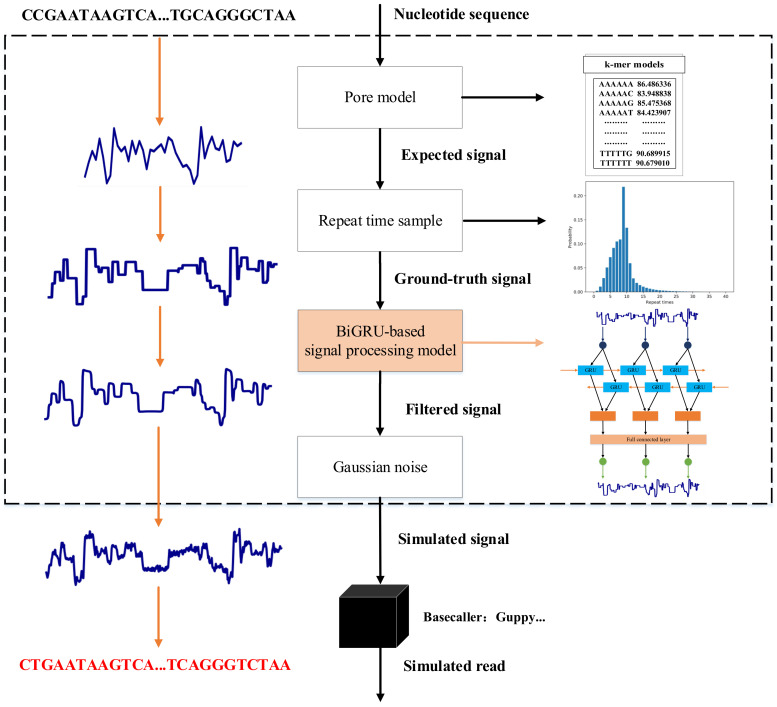
The main workflow of the proposed signal simulation method based on BiGRU (NanosigSim). Given an input nucleotide sequence, this method first generates the expected signal by the pore model. Then, the expected signal is used to produce the ground-truth signal according to the repeat length distribution. Finally, to simulate the real-world sequencing signal, it applies BiGRU-based signal processing model and adds the Gaussian noise on the ground-truth signal.

Based on the research of DeepSimulator, we propose a novel nanopore sequencing signal simulation method based on BiGRU. DeepSimulator is the first simulator that completely simulates the entire pipeline of nanopore sequencing, which can generate simulated signals and simulated reads simultaneously. The nanopore sequencing pipeline mainly includes three stages: sample preparation, current signal collection and basecalling [19]. Correspondingly, the main workflow of DeepSimulator consists of a sequence generator module, a signal generator module and a basecaller module. The sequence generator module randomly selects the starting position on the input reference genome sequence to generate relatively short sequences that satisfy the length distribution of real sequencing reads. The signal generator module generates the simulated signals corresponding to the nucleotide sequences output by the previous module according to the nanopore sequencing 6-mer pore model. The final basecaller module translates the simulated signals into simulated reads [20]. Our proposed signal simulation method mainly improves the signal generator module of DeepSimulator. The main workflow of our proposed signal simulation method is shown in Figure 1, which can simulate the process of measuring the electrical current signal of an input nucleotide sequence using a nanopore sequencer (such as MinION).

The nanopore sequencing signal is mainly influenced by the 5 or 6 bases occupying the pore at the same time. The 6-mer pore model provided by ONT contains the expected current signal value corresponding to each 6-mer [21]. Given an input nucleotide sequence $X=x_{1},x_{2},\ldots ,x_{T}$ with *T* bases, the first step in the process of outputting its corresponding simulated signal is to convert it into the expected signal sequence $Y=y_{1},y_{2},\ldots ,y_{T-5}$ via the pore model, where $y_{i}$ represents the corresponding expected signal value of the 6-mer starting from position *i* in *X*.

With the current MinION pore chemistry, ONT reports that the single-stranded nucleotide sequence passes through the pore at a speed around 450 bp/s, and the sampling frequency of sequencing signal is 4 kHz. Thus, there are on average 8–9 discrete measurements per 6-mer, although the number varies because of the fluctuating translocation speed of the motor protein. To convert the expected signal sequence to the electrical current signal sequence which can be put into a basecaller, we need to repeat each signal value several times in the expected signal sequence. The repeat time of expected signal is obtained from the real-world experimental data, which satisfies the mixture alpha distribution. The signal sequence generated by the above process is called ground-truth signal, and the signal has a similar length distribution with the real sequencing signal.

The simulated ground-truth signal is composed of a series of square waves, whose spectrum is the combination of infinite sine waves. To simulate the nanopore sequencing signal more realistically, it is necessary to filter the high-frequency components embedded in the square waves. DeepSimulator uses a low-pass filter which is realized by convoluting the ground-truth signal with a windowed-sinc function to achieve this process. Considering that the speed of single-stranded nucleotide sequence passing through the nanopore is around 450 bp/s, the cutoff frequency of the low-pass filter should be larger than 450 Hz. When the cutoff frequency is set to 950 Hz, the simulated signal which is most similar to the real signal can be generated. Different from DeepSimulator, we build a signal processing model based on BiGRU to process the ground-truth signal. The proposed signal processing model can model the relation between ground-truth signal and real-world sequencing signal through experimental data to accurately filter out the useless high-frequency components. This design can greatly improve the simulation accuracy of nanopore sequencing signal. The specific implementation details of the proposed signal processing model are described below.

Compared with the real-world sequencing signal, the filtered signals output by the low-pass filter and the proposed signal processing model do not contain any noise characteristics, while the complex sequencing environment will output the real sequencing signal with a low signal-to-noise ratio. By adding Gaussian noise to each position of the simulated signal, the complex sequencing environment can be simulated. Changing the variance of the Gaussian noise added later can effectively control the quality of the output simulated signal.

### 2.2. Gated Recurrent Units Neural Network

GRU is a popular variant of recurrent neural network, which can effectively solve the gradient problem during back propagation through the network [22]. Compared with long short-term memory (LSTM) [23], another variant of recurrent neural network, GRU not only inherits the gate control principle of LSTM, but also simplifies the structure of neurons and reduces the complexity of the model. GRU has the advantages of fewer parameters, simpler structure, easier computation, and stronger convergence.

**Figure 2:**
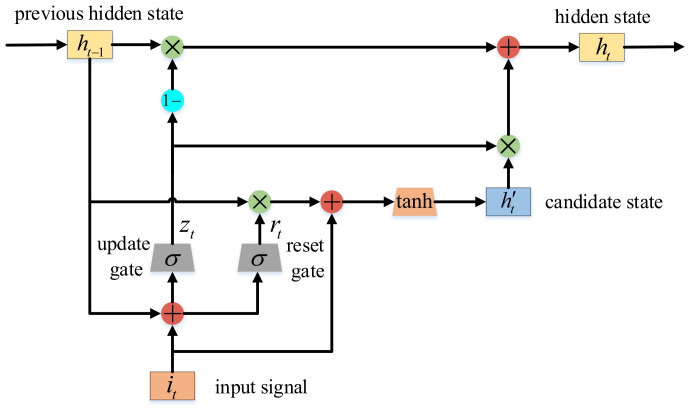
The specific structure of GRU neural network.

The GRU network structure includes two gates and two states, which are update gate *z*, reset gate *r*, hidden state *h*, and candidate state $h^{\prime }$. In Figure 2, $i_{t}$ represents the value of the input signal at time *t*, $h_{t-1}$ represents the hidden state at the previous time, and the hidden state $h_{t}$ at time *t* is determined by $i_{t}$ and $h_{t-1}$. Given the input signal $i_{t}$ and hidden state $h_{t-1}$, GRU first calculates update gate $z_{t}$ and reset gate $r_{t}$, which control how to get $h_{t}$ from $i_{t}$ and $h_{t-1}$. In this structure, the update gate $z_{t}$ is calculated by Equation (1) and the reset gate $r_{t}$ is calculated by Equation (2), where σ is the sigmoid function $\sigma \left(z\right)=1/\left(1+e^{-z}\right)$; $W_{z}$ and $W_{r}$ are the update gate weight and reset gate weight respectively; and $b_{z}$ and $b_{r}$ are biases.

(1) $$z_{t}=\sigma \left(W_{z}\left[h_{t-1},i_{t}\right]+b_{z}\right)$$

(2) $$r_{t}=\sigma \left(W_{r}\left[h_{t-1},i_{t}\right]+b_{r}\right)$$

Then, the unit computes the candidate state $h_{t}^{\prime }$ using the reset gate $r_{t}$, i.e.,

(3) $$h_{t}^{\prime }=\tanh\left(W\left[r_{t}\circ h_{t-1},i_{t}\right]\right)$$

where ∘ represents the element-wise vector product and *W* represents the weight matrix. If the component of the reset gate vector is close to 0, it will reduce the impact of the previous state. Finally, the overall output $h_{t}$ of GRU network is a linear combination of $h_{t}^{\prime }$ and $h_{t-1}$, i.e.,

(4) $$h_{t}=\left(1-z_{t}\right)\circ h_{t-1}+z_{t}\circ h_{t}^{\prime }$$

### 2.3. Proposed Signal Processing Model Based on BiGRU

To more accurately simulate the real-world nanopore sequencing signals, a key step in DeepSimulator’s signal generator module is using a low-pass filter to post-process the ground-truth signals. A low-pass filter is a filter that passes signals with a frequency lower than the selected cutoff frequency and attenuates signals with a frequency higher than the cutoff frequency. Although the low-pass filter can filter out high-frequency components that are not related to the real signals, it also filters out high-frequency components related to the real signals, resulting in a large distortion between the simulated signals and the real signals. To solve this problem, we build a signal processing model based on BiGRU to post-process the ground-truth signals. The proposed model can learn how to filter out the irrelevant high-frequency components accurately from the experimental data and retain the useful high-frequency components, generating simulated signals closer to the real-world signals.

**Figure 3:**
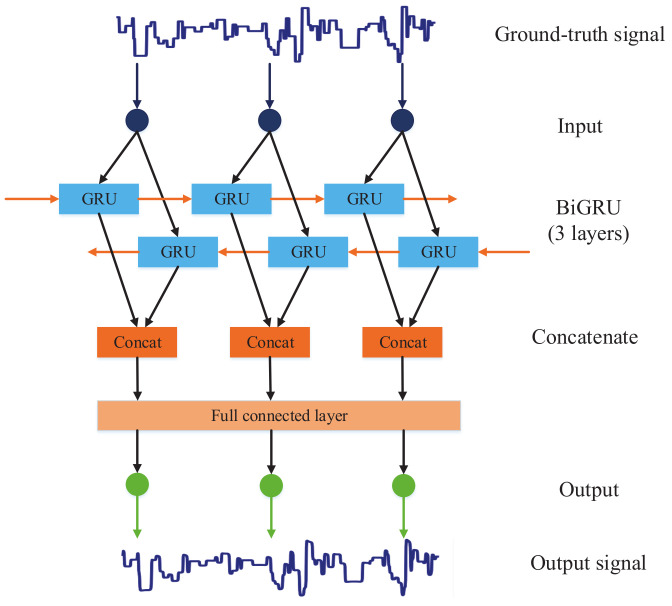
The network architecture of signal processing model based on BiGRU, which is composed of a three-layer BiGRU and a fully connected layer. Given a ground-truth signal, the signal processing model will output its corresponding filtered signal after calculation.

The network architecture of the signal processing model based on BiGRU proposed in this study is shown in Figure 3. This model combines a three-layer BiGRU and a fully connected layer to post-process the ground-truth signals. The normalized ground-truth signal $I=i_{1},i_{2},\ldots ,i_{T}$ is used as the input of the model and the output of the model is the filtered signal $O=o_{1},o_{2},\ldots ,o_{T}$ with the same length as the input signal. The final simulated signal is generated by adding Gaussian noise to the output signal.

**Figure 4:**
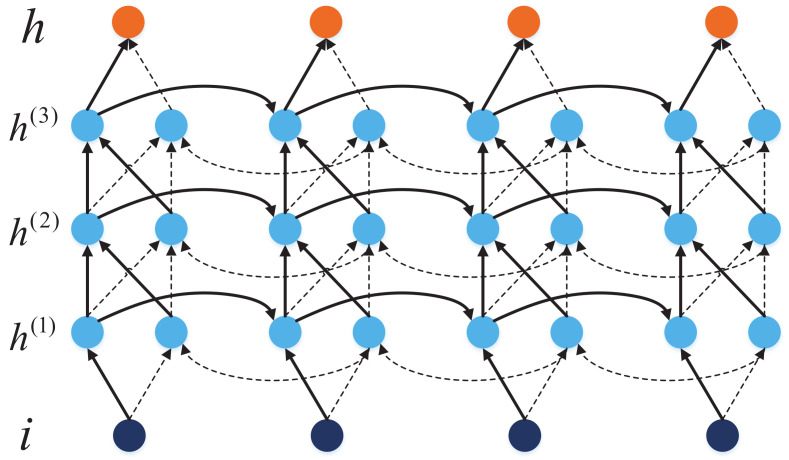
The structure of the three-layer BiGRU network.

The core module of the above ground-truth signal processing model is the BiGRU network. When using the GRU network to process the ground-truth signal, the output signal value $o_{t}$ at the time *t* is not only related to the present input signal value, but also related to the input signal values previous and afterward. To connect the output value at the current time with the state at the time before and after, the ground-truth signal is processed by the BiGRU network, and the output signal value $o_{t}$ is more accurate through forward and backward calculation. The basic unit of the BiGRU model consists of a forward-propagating GRU unit and a backward-propagating GRU unit. The hidden state $h_{t}$ at time *t* is obtained by concatenating the forward hidden state $h_{t}^{f}$ and the backward hidden state $h_{t}^{r}$. The specific calculation process is as follows:

(5) $$h_{t}^{f}=GRU\left(h_{t-1}^{f},i_{t}\right)$$

(6) $$h_{t}^{r}=GRU\left(h_{t+1}^{r},i_{t}\right)$$

(7) $$h_{t}=h_{t}^{f}\left|\right|h_{t}^{r}$$

where || denotes concatenation of vectors. The signal processing model proposed in this study adopts three-layer BiGRU. Using multi-layer BiGRU can increase the parameters of the model and improve the learning ability of the model. Figure 4 shows the structure diagram of the three-layer BiGRU network used in this paper.

The output vector of the last layer of the three-layer BiGRU network is then fed into the fully connected layer, and the output of the fully connected layer is the final filtered signal. The novelty of our proposed model is to use neural networks to learn the relationship between ground-truth signals and corresponding real-world sequencing signals. Compared with the low-pass filter of the DeepSimulator’s signal generator module, the proposed signal processing model can efficiently and accurately filter out the useless high-frequency components embedded in the ground-truth signal, generating simulated signal which is more similar to the real-world signal in the time domain and frequency domain.

### 2.4. Parameter Training of Proposed Signal Processing Model

To make the proposed signal processing model able to process the ground-truth signal correctly, it is necessary to use training data to train the parameters of the model. Through parameter training, the model can learn the internal relationship between the ground-truth signal and the real sequencing signal which can achieve efficient and accurate signal filtering. The parameter training process of the signal processing model mainly includes four major steps: preparing supervised training data, defining the loss function, initializing the model parameters, and iterating the model parameters using the optimization algorithm.

The first step of parameter training is the preparation of the supervised training data in advance. In this model, the input vector is the ground-truth signal calculated by the nucleotide sequence and 6-mer pore model, and the output vector is the filtered signal with high-frequency characteristics similar to the real sequencing signal. Therefore, the supervised training data should include the real sequencing signals and the corresponding ground-truth signals. The sequencing signal generated by MinION sequencing platform [24] of ONT is stored in fast5 file [25], which is an HDF5 file format. In this study, the sequencing signal is extracted by h5py library of Python. After getting the real sequencing signal, the key to preparing supervised training data is to calculate the ground-truth signal corresponding to the sequencing signal. This process can be achieved by using continuous wavelet dynamic time warping (cwDTW) to label the sequencing signal [26].

**Figure 5:**
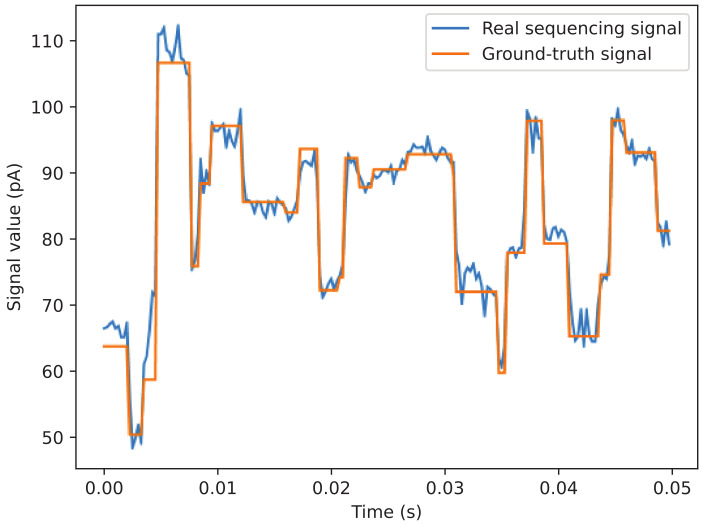
The supervised training data of signal processing model, including the real sequencing signal and the corresponding ground-truth signal. The ground-truth signal is calculated by labeling the real sequencing signal.

Given the sequencing signal sequence and the reference genome sequence, the goal of signal labeling is to linearly map each position on the sequencing signal sequence to a corresponding base on the genome sequence. Firstly, the sequencing signal sequence is translated to a sequencing read which is then aligned with the reference genome using the gene sequence alignment algorithm [27,28]. Then, according to the results of alignment, the expected signal sequence can be calculated based on genomic sequence fragment and the known 6-mer pore model. Finally, cwDTW algorithm is utilized to complete the end-to-end mapping between the sequencing signal sequence and the expected signal sequence. As shown in Figure 5, according to the mapping results, the expected signal value corresponding to each position in the sequencing signal sequence can be obtained, and the corresponding ground-truth signal is then outputted.

In addition to preparing supervised training data in advance, another key to parameter training is to define the loss function of the model. Suppose the input ground-truth signal of model is $I=i_{1},i_{2},\ldots ,i_{T}$, its corresponding real sequencing signal is $R=r_{1},r_{2},\ldots ,r_{T}$, and the output signal of signal processing model is $O=o_{1},o_{2},\ldots ,o_{T}$. The loss function used in this study is the Log-Cosh loss function, that is

(8) $$L\left(R,O\right)=\sum_{i=1}^{T}\log\left(\cosh\left(o_{i}-r_{i}\right)\right)$$

After the definition of the model’s loss function, the model parameters are initialized. The initialization of neural network parameters is an important basic part of training process, which will have an important impact on the performance and convergence speed of the model. We use the Xavier initialization method to initialize the model parameters, that is, the model parameters are initialized to a uniform distribution within the interval $\left[-\sqrt{\frac{6}{n_{i}+n_{o}}},\sqrt{\frac{6}{n_{i}+n_{o}}}\right]$, where $n_{i}$ represents the input vector dimension of each layer of the network and $n_{o}$ represents the output vector dimension.

**Figure 6:**
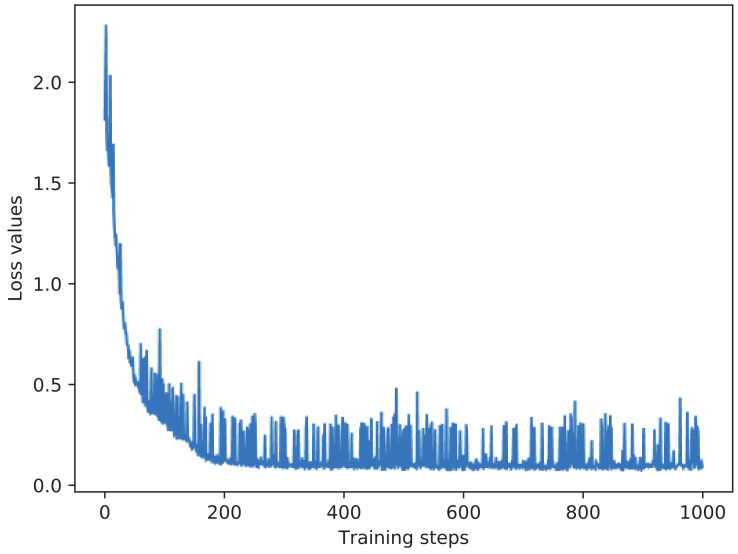
The loss value change with respect to the training iteration steps.

The goal of the parameter training is to find the parameters of the network that minimize the loss function, which can be achieved by applying an optimization algorithm to iterate parameters. We choose the Adam adaptive optimizer with the learning rate of 0.0001 to minimize the Log-Cosh loss to achieve parameter training, which is a popular adaptive optimization algorithm. We set the batch size as 128 and the number of iterations as 1000 during training. The proposed signal processing model based on BiGRU is implemented using Tensorflow [29]. The training process of the model is illustrated in Figure 6, which shows the loss value change with respect to the training iteration steps.

### 2.5. Datasets

We have used the existing datasets sequenced on MinION R9.4 flowcells to train and evaluate the performance of the proposed signal simulation method [30]. Specifically, the training dataset consists of 4000 Lambda virus reads and 4000 *Escherichia coli* reads (http://gigadb.org/dataset/100425), and the test dataset consists of 4467 Acinetobacter pittii reads, 15,178 Klebsiella pneumoniae reads, 16,742 Serratia marcescens reads, and 11,047 Staphylococcus aureus reads (https://bridges.monash.edu/articles/Raw_fast5s/7676174).

### 2.6. Analysis Methods of Simulation Results

We use DeepSimulator1.5 and the proposed BiGRU-based simulation method to generate the simulated signals corresponding to each real sequencing signal in the test dataset and compare the two simulated signals from multiple aspects. For DeepSimulator, the cutoff frequency was set to 950 Hz, the mean value of the Gaussian noise was set to 0 pA, and the standard deviation of the Gaussian noise was set to 2.0. The standard deviation of the Gaussian noise in our simulation method was also set to 2.0 for fair comparison. The noise level of DeepSimulator and our method was the same. In the two signal simulation methods, the added Gaussian noise was random noise not correlated in time. Firstly, the two different filtered signal waveforms, which are output by the signal processing model based on BiGRU and the low-pass filter respectively, were compared. Then we employed dynamic time warping (DTW) algorithm [31] and continuous wavelet transform (CWT) algorithm to evaluate the similarity between the two simulated signals and the real sequencing signals. Finally, the error characteristics of the three sequencing reads generated from the simulated signals and the real sequencing signals were analyzed.

## 3. Results and Discussion

### 3.1. Waveform Comparison of Two Filtered Signals

**Figure 7:**
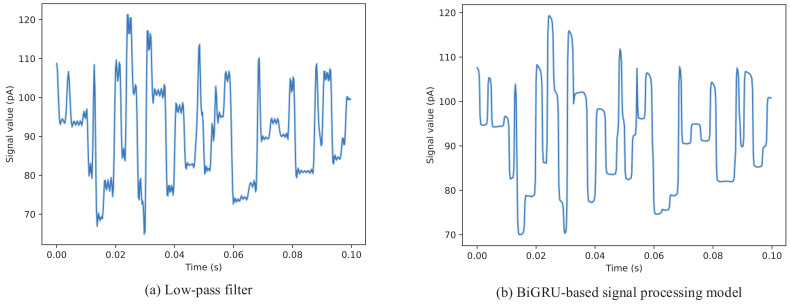
Waveform comparison of two filtered signals from different signal processing methods: (**a**) the filtered signal output by low-pass filter; and (**b**) the filtered signal output by BiGRU-based signal processing model. Compared with low-pass filter, BiGRU-based signal processing model only processes the signal at the 6-mer change, which is more reasonable.

To verify whether the proposed BiGRU-based signal processing model can efficiently and accurately filter out the useless high-frequency components embedded in the ground-truth signal, the low-pass filter of the DeepSimulator’s signal generator module and the proposed signal processing model were used to process the ground-truth signal independently. The comparison results of the filtered signal waveforms are shown in Figure 7. In general, although the two filtered signals are slightly different in specific details, they both retain the main characteristics of the ground-truth signal. According to the signal waveforms, it can be observed that, compared with the traditional low-pass filter, the signal processing model based on BiGRU only filters the signal at the 6-mer change in the ground-truth signal, which is more in line with the real nanopore sequencing data. The comparison results indicate that the proposed model can accurately learn the internal relationship between the ground-truth signal and the real sequencing signal.

### 3.2. Analysis of Simulated Signals Using DTW Algorithm

**Figure 8:**
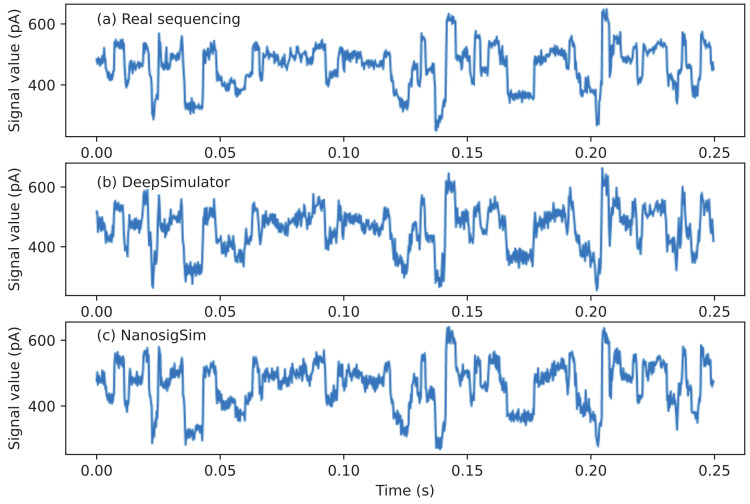
The comparison between: (**a**) the real sequencing signal waveform; (**b**) the simulated signal waveform from DeepSimulator; and (**c**) the simulated signal waveform from NanosigSim. The simulated signals from both DeepSimulator and NanosigSim are similar to the real sequencing signal.

**Figure 9:**
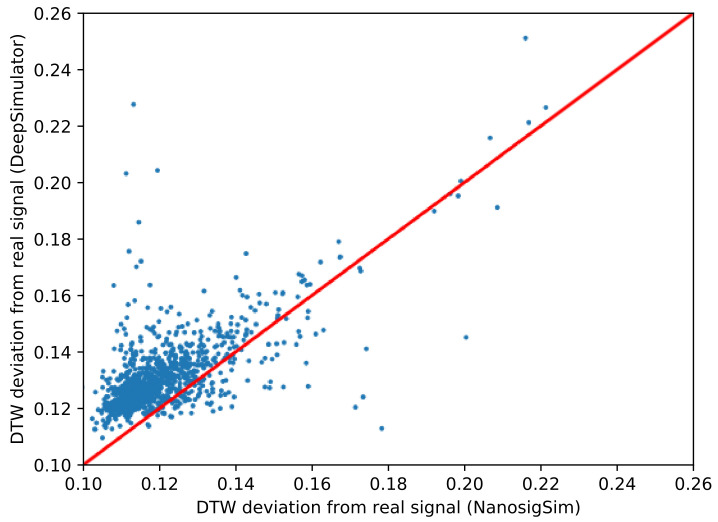
The similarity comparison between the two simulated signals and the real sequencing signal based on DTW distance. Any point above the red line means our simulation is better, whereas any point below means DeepSimulator is better.

The final simulated signals can be generated by adding Gaussian noise to the filtered signals. As shown in Figure 8, both DeepSimulator and NanosigSim can generate the simulated signals similar to the real sequencing signal waveform. To quantitatively analyze the similarity between the two simulated signals and the real sequencing signal, we employed DTW algorithm for signal analysis, which is a standard method to check the difference between two signals. We tested the performance on the randomly selected 1000 sequencing samples in the test dataset. The average normalized DTW distance between the simulated signals generated by NanosigSim and the real sequencing signals is 0.121, which is about 7.5% lower than that of DeepSimulator (0.132). This indicates that the proposed method can generate the simulated signals closer to the real sequencing signals. Figure 9 shows the experimental results of 1000 sequencing samples. Each point in the figure represents a sequencing sample and the red line is the diagonal line. The points above the red line mean NanosigSim is better, while the points below the red line mean DeepSimulator is better. According to the statistical results, about 89.1% of the test samples show that NanosigSim has higher accuracy.

### 3.3. Analysis of Simulated Signals Using CWT Algorithm

**Figure 10:**
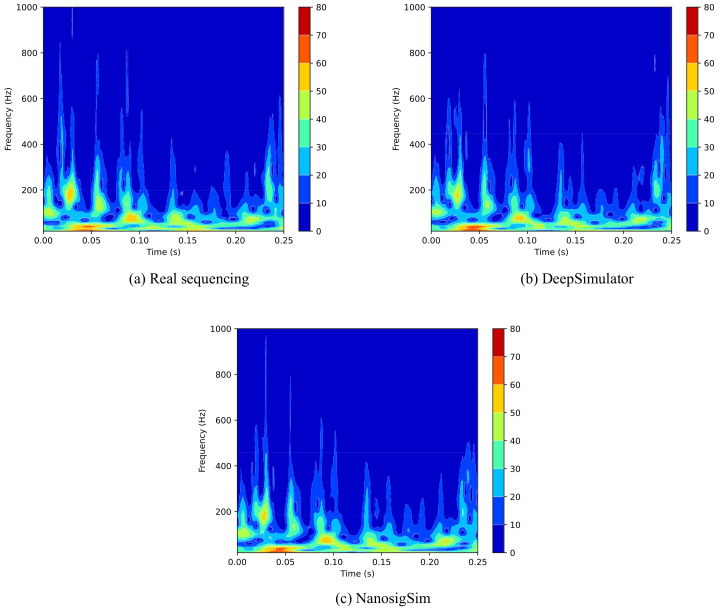
The CWT spectrums of: (**a**) the real sequencing signal; (**b**) the simulated signal from DeepSimulator; and (**c**) the simulated signal from NanosigSim. The CWT spectrum for NanosigSim is more similar to the spectrum for the real signal than that for DeepSimulator, which means that our proposed method is better.

In addition to comparing the DTW distance between the simulated signals and the real sequencing signals, we also used CWT algorithm to perform time–frequency analysis on the three signals. The CWT spectrum of each signal is depicted in Figure 10. Overall, the CWT spectrums of the two simulated signals are very similar to that of real sequencing signal, but the spectrum of the simulated signal generated by NanosigSim is more similar to the spectrum of real sequencing signal, especially the high-frequency part. To quantitatively measure the similarity between the simulated signals and the real signal in terms of the CWT spectrum, we calculated the Pearson correlation coefficient (PCC) between the spectrums of two simulated signals and the spectrum of the real signal and further calculated the PCC in the low-frequency and high-frequency parts of the CWT spectrum. The PCC is a standard method used to calculate the correlation between two CWT spectrums. The calculation results are shown in Table 1.

Table 1 shows that the PCC between the spectrum of the simulated signal generated by NanosigSim and the spectrum of real sequencing signal is improved by 9.08% compared with DeepSimulator. Moreover, the improvement of PCC in the high-frequency part is even higher (18.88%), which further suggests that the high-frequency details processed by the signal processing model based on BiGRU are indeed consistent with the real sequencing signal and the proposed simulation method effectively improves the signal simulation accuracy.

### 3.4. Analysis of Simulated Reads

**Figure 11:**
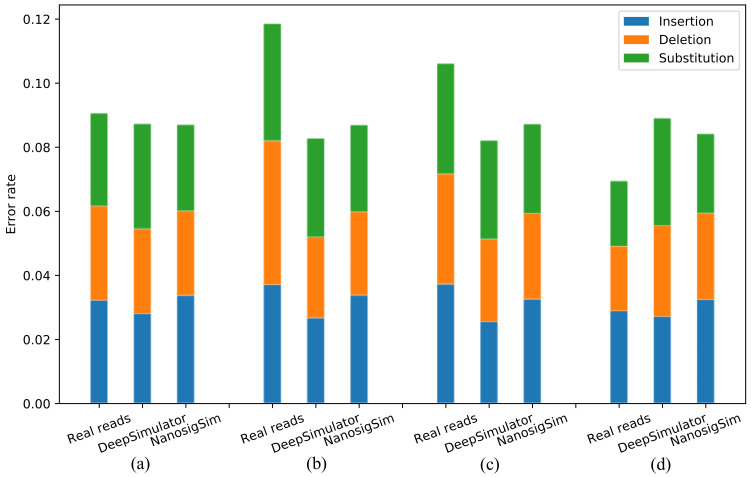
The profiles of different types of reads, tested on the four biological samples provided by the test dataset, which are basecalled using Guppy: (**a**) Acinetobacter pittii sample; (**b**) Klebsiella pneumoniae sample; (**c**) Serratia marcescens sample; and (**d**) Staphylococcus aureus sample.

An important indicator for evaluating the quality of simulated signals is to compare whether the simulated reads generated by the simulated signals have similar error characteristics to the real sequencing reads. We used Guppy, the newest official basecaller, to basecall the two simulated signals and the real sequencing signals, respectively. Then, edlib software [32] was adopted to calculate the error characteristics of simulated reads and real sequencing reads, including insertion rate, deletion rate, and substitution rate. The statistical results obtained on the four biological samples provided by the test dataset are shown in Figure 11. Since many factors such as the sequencing biological species and the experimental purpose will influence the read accuracy, the error rates of the four real sequencing reads are quite different. The two simulated reads generated by DeepSimulator and NanosigSim have similar error rates. However, in terms of the specific distribution of the three types of errors, the two simulated reads are not similar. For the three errors in the simulated reads generated by DeepSimulator, substitution accounts for the largest proportion, followed by insertion and deletion. Among the errors in our simulated reads, insertion accounts for the most, followed by substitution and deletion.

To compare the error characteristics of the two simulated reads and the four real reads in detail, we further calculated the proportions of the three types of errors in the six reads. As shown in Table 2, except for the Klebsiella pneumoniae sample, the simulated reads generated by NanosigSim have a similar error distribution to the real sequencing reads. However, the difference between the simulated reads generated by DeepSimulator and the real sequencing reads is large in terms of the error distribution for all biological samples, which may be caused by inaccurate filtering of the ground-truth signal by the low-pass filter. The analysis results of the simulated reads show that the nanopore sequencing signal simulation method proposed in this paper greatly improves the quality of simulated signals.

### 3.5. Custom Model for Klebsiella Pneumoniae

**Figure 12:**
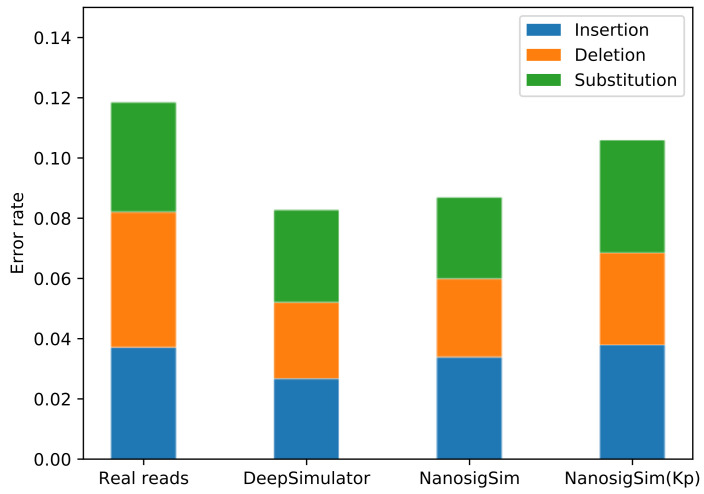
The profiles of different types of reads, tested on the Klebsiella pneumoniae sample, which are basecalled using Guppy. NanosigSim (Kp) means using the custom model for Klebsiella pneumoniae to generate simulated reads. Compared with the other two simulated reads, the simulated reads generated by NanosigSim (Kp) are more similar to the real reads in terms of error characteristics.

For the Klebsiella pneumoniae sample, the two simulated reads generated by DeepSimulator and NanosigSim are both quite different from the real sequencing reads. To further explore the scalability of NanosigSim, we randomly selected 4000 sequencing reads from the Klebsiella pneumoniae sample to train the BiGRU-based signal processing model, and the remaining sequencing reads were used as the test dataset to verify the performance of the custom model for Klebsiella pneumoniae. As shown in Figure 12, the simulated reads generated by the custom model for Klebsiella pneumoniae have similar insertion rate and substitution rate as the real sequencing reads, while the deletion rate of the simulated reads is smaller than that of the real sequencing reads. Compared with the other two simulated reads, the error characteristics of the simulated reads generated by the custom model for Klebsiella pneumoniae are closer to the real sequencing reads, which indicates that NanosigSim can effectively learn the time–frequency characteristics of real sequencing signals. The simulation results show that, compared with DeepSimulator, an obvious advantage of our simulation method is that it can learn the corresponding time–frequency characteristics according to the provided training data generated by different sequencing experiments.

## 4. Conclusions

In this paper, we propose a novel signal simulation method of nanopore sequencing based on BiGRU. To improve the simulation accuracy of existing methods, we build a signal processing model based on BiGRU to process the ground-truth signal calculated by the input nucleotide sequence and nanopore sequencing pore model. Compared with low-pass filter, the proposed model can effectively filter out unwanted high-frequency components embedded in the ground-truth signal and retain the useful high-frequency components by using the efficient self-learning ability of the BiGRU neural network. The simulation results show that the proposed method can provide high-quality simulated data for the methods and tools developed for the applications of nanopore sequencing. Although the proposed method can effectively improve the simulation accuracy, it inevitably requires more computational resources. In the future, further structure optimization of the proposed signal processing model is required to reduce the complexity of the signal simulation method.

## Figures and Tables

**Table 1:** PCC comparison between the CWT spectrums of different signals.

	PCC (All)	PCC (Low-Frequency)	PCC (High-Frequency)
DeepSimulator	0.837	0.939	0.715
NanosigSim	0.913	0.964	0.850
Improvement	9.08%	2.27%	18.88%

**Table 2:** The error distribution of different types of reads, tested on the four real reads and the two simulated reads, which are basecalled using Guppy.

	Insertion	Deletion	Substitution
Acinetobacter pittii	35.54%	32.45%	32.01%
Klebsiella pneumoniae	31.31%	37.89%	30.80%
Serratia marcescens	35.06%	32.52%	32.42%
Staphylococcus aureus	41.64%	29.11%	29.25%
DeepSimulator	32.07%	30.36%	37.57%
NanosigSim	38.74%	30.04%	31.22%

## References

[1] Deamer D., Akeson M., Branton D. (2016). Three decades of nanopore sequencing. Nat. Biotechnol..

[2] Leggett R.M., Clark M.D. (2017). A world of opportunities with nanopore sequencing. J. Exp. Bot..

[3] Rang F.J., Kloosterman W.P., de Ridder J. (2018). From squiggle to basepair: Computational approaches for improving nanopore sequencing read accuracy. Genome Biol..

[4] Cherf G.M., Lieberman K.R., Rashid H., Lam C.E., Karplus K., Akeson M. (2012). Automated forward and reverse ratcheting of DNA in a nanopore at 5-angstrom precision. Nat. Biotechnol..

[5] Byrne A., Beaudin A.E., Olsen H.E., Jain M., Cole C., Palmer T., DuBois R.M., Forsberg E.C., Akeson M., Vollmers C. (2017). Nanopore long-read RNAseq reveals widespread transcriptional variation among the surface receptors of individual B cells. Nat. Commun..

[6] Faria N.R., Sabino E.C., Nunes M.R.T., Alcantara L.C., Loman N.J., Pybus O.G. (2016). Mobile real-time surveillance of Zika virus in Brazil. Genome Med..

[7] Simpson J.T., Workman R.E., Zuzarte P.C., David M., Dursi L.J., Timp W. (2017). Detecting DNA cytosine methylation using nanopore sequencing. Nat. Methods.

[8] Arima A., Harlisa I.H., Yoshida T., Tsutsui M., Tanaka M., Yokota K., Tonomura W., Yasuda J., Taniguchi M., Washio T. (2018). Identifying single viruses using biorecognition solid-state nanopores. J. Am. Chem. Soc..

[9] Varongchayakul N., Song J.X., Meller A., Grinstaff M.W. (2018). Single-molecule protein sensing in a nanopore: A tutorial. Chem. Soc. Rev..

[10] Chinappi M., Cecconi F. (2018). Protein sequencing via nanopore based devices: A nanofluidics perspective. J. Phys. Condes. Matter.

[11] Fragasso A., Schmid S., Dekker C. (2020). Comparing current noise in biological and solid-state nanopores. ACS Nano.

[12] Wee Y., Bhyan S.B., Liu Y.N., Lu J.C., Li X.Y., Zhao M. (2019). The bioinformatics tools for the genome assembly and analysis based on third-generation sequencing. Brief. Funct. Genom..

[13] Li Y., Huang C., Ding L.Z., Li Z.X., Pan Y.J., Gao X. (2019). Deep learning in bioinformatics: Introduction, application, and perspective in the big data era. Methods.

[14] Makalowski W., Shabardina V. (2020). Bioinformatics of nanopore sequencing. J. Hum. Genet..

[15] Escalona M., Rocha S., Posada D. (2016). A comparison of tools for the simulation of genomic next-generation sequencing data. Nat. Rev. Genet..

[16] Yang C., Chu J., Warren R.L., Birol I. (2017). NanoSim: Nanopore sequence read simulator based on statistical characterization. GigaScience.

[17] Li Y., Han R.M., Bi C.W., Li M., Wang S., Gao X. (2018). DeepSimulator: A deep simulator for Nanopore sequencing. Bioinformatics.

[18] Li Y., Wang S., Bi C.W., Qiu Z.W., Li M., Gao X. (2020). DeepSimulator1.5: A more powerful, quicker and lighter simulator for Nanopore sequencing. Bioinformatics.

[19] Loman N.J., Quick J., Simpson J.T. (2015). A complete bacterial genome assembled de novo using only nanopore sequencing data. Nat. Methods.

[20] Wick R.R., Judd L.M., Holt K.E. (2019). Performance of neural network basecalling tools for Oxford Nanopore sequencing. Genome Biol..

[21] David M., Dursi L.J., Yao D.L., Boutros P.C., Simpson J.T. (2017). Nanocall: An open source basecaller for Oxford Nanopore sequencing data. Bioinformatics.

[22] Boza V., Brejova B., Vinar T. (2017). DeepNano: Deep recurrent neural networks for base calling in MinION nanopore reads. PLoS ONE.

[23] Shi B.G., Bai X., Yao C. (2017). An end-to-end trainable neural network for image-based sequence recognition and its application to scene text recognition. IEEE Trans. Pattern Anal. Mach. Intell..

[24] Jain M., Olsen H.E., Paten B., Akeson M. (2016). The Oxford Nanopore MinION: Delivery of nanopore sequencing to the genomics community. Genome Biol..

[25] Payne A., Holmes N., Rakyan V., Loose M. (2019). BulkVis: A graphical viewer for Oxford nanopore bulk FAST5 files. Bioinformatics.

[26] Han R.M., Li Y., Gao X., Wang S. (2018). An accurate and rapid continuous wavelet dynamic time warping algorithm for end-to-end mapping in ultra-long nanopore sequencing. Bioinformatics.

[27] Li H. (2016). Minimap and miniasm: Fast mapping and de novo assembly for noisy long sequences. Bioinformatics.

[28] Li H. (2018). Minimap2: Pairwise alignment for nucleotide sequences. Bioinformatics.

[29] Abadi M. Tensorflow: Learning functions at scale. Proceedings of the 21st ACM SIGPLAN International Conference on Functional Programming.

[30] Teng H.T., Cao M.D., Hall M.B., Duarte T., Wang S., Coin L.J.M. (2018). Chiron: Translating nanopore raw signal directly into nucleotide sequence using deep learning. GigaScience.

[31] Salvadora S., Chan P. (2007). Toward accurate dynamic time warping in linear time and space. Intell. Data Anal..

[32] Sosic M., Sikic M. (2017). Edlib: A C/C plus plus library for fast, exact sequence alignment using edit distance. Bioinformatics.

